# *Neurology International* Expands Its’ Scope and Mission: Exploring the Nervous System in Health and Disease with Emphasis on Molecular and Pathologic Aspects

**DOI:** 10.3390/neurolint12030013

**Published:** 2020-11-18

**Authors:** Michael A. Meyer

**Affiliations:** Invision Health, Department of Neurology, International Drive, Williamsville, NY 14221, USA; michaelandrewmeyer@gmail.com

Having served as editor-in-chief for *Neurology International* since its inception back in 2009, I am very pleased to see the journal grow and expand, with a major development now taking place with new ownership by MDPI, a world leader in Open Access Journal publication. I look forward to working with our staff and Editorial Board in elevating the quality and volume of publications that ultimately are accepted, after rigorous peer review by two or more experts has been completed. Our scope continues to be broad, encompassing both clinical and basic science aspects of the nervous system; in particular, we encourage submissions dealing with the molecular and pathological aspects of the nervous system in health and disease. We encourage both clinical and basic science-oriented submissions to keep up with our rapidly expanding knowledge of the human genome (and in particular the “brain proteome”—please follow this link for more information: https://www.proteinatlas.org/humanproteome/brain).

MDPI is quite an amazing partner to work with, as they currently publish 281 other journals besides *Neurology International.* Related MDPI journals include *Audiology Research* [1], *Brain Sciences* [2], *Neuroglia* [3], *Neurosci* [4].

Topics of special interest will be announced far enough in advance for the preparation of high quality submissions for prompt peer review. We hope to publish special issues that address “hot topics” in multiple areas that include but are not limited to Parkinson’s disease, stroke, Alzheimer disease, epilepsy, multiple sclerosis, and other disorders of the brain and nervous system, and invite all interested authors to make preparations for this now.

Michael A. Meyer, MD

Editor-in-Chief for *Neurology International*

**Short Biography**


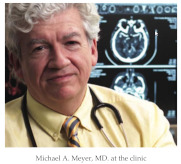


Dr. Mike Meyer trained as a physician at Mayo Clinic with dual board certifications in Neurology and Nuclear Medicine. Prior to medical school at Albany Medical College, Dr. Meyer was part of the original research team at Brookhaven National Lab that developed 18F-FDG as a useful tracer for PET studies in Oncology and Neurology [5]. After completing fellowships in PET and Nuclear Medicine at University of Michigan and University of Pennsylvania, he represented the University of Tennessee as one of eight Principal Investigators for the NINDS Acute Stroke tPA trial and was co-author on the landmark December 1995 publication on tPA and Stroke in the *New England Journal of Medicine* [6]. Dr. Meyer has trained many neurology residents during his career as a neurologist, and had served as the residency director for the University at Buffalo; other related accomplishments include writing a single author textbook of neurology published by Springer [7].

Dr. Meyer has extensive experience in the development and administration of regional stroke centers specializing in managing acute ischemic stroke; examples of centers he helped establish include ECMC in Buffalo and Tennova Healthcare in Knoxville, TN. Dr. Meyer is currently with Invision Health, Department of Neurology, International Drive, Williamsville, New York, and maintains strong research interests in Alzheimer’s disease [8,9]; he very much looks forward to working with MDPI to develop *Neurology International*.
